# The Role of Caspase-1 and Caspase-4 in Modulating Gingival Epithelial Cell Responses to *Aggregatibacter actinomycetemcomitans* Infection

**DOI:** 10.3390/pathogens14030295

**Published:** 2025-03-18

**Authors:** Kartheyaene Jayaprakash Demirel, Alessandra Neves Guimaraes, Isak Demirel

**Affiliations:** 1 Department of Oral and Maxillofacial Surgery, Faculty of Medicine and Health, Örebro University, 701 82 Örebro, Sweden; 2 Department of Odontological Research, Public Dental Service, Faculty of Medicine and Health, Örebro University, 701 82 Örebro, Sweden; alessandra.neves-guimaraes@regionorebrolan.se; 3 Department of Periodontology and Implantology, Public Dental Service, Faculty of Medicine and Health, Örebro University, 701 82 Örebro, Sweden; 4 School of Medical Sciences, Örebro University, 701 82 Örebro, Sweden; isak.demirel@oru.se

**Keywords:** *Aggregatibacter actinomycetemcomitans*, caspase-1, caspase-4, periodontitis, gingival epithelial cells

## Abstract

Periodontitis is a chronic inflammatory disease characterized by bacterial infection and immune dysregulation. *Aggregatibacter actinomycetemcomitans* (*A. actinomycetemcomitans*) is a key pathogen linked to disease progression. Caspase-1 and caspase-4 regulate inflammasome activation and cytokine release, yet their roles in gingival epithelial immunity remain unclear. The aim of this study was to elucidate the involvement of caspase-1 and caspase-4 in regulating the immune response to *A. actinomycetemcomitans* infection in gingival epithelial cells. Human gingival epithelial cells (Ca9-22) and caspase-1- and caspase-4-deficient cells were infected with *A. actinomycetemcomitans* for 24 h. Inflammatory mediator release was analyzed using Olink proteomics. Bacterial colonization and invasion were assessed using fluorescence-based assays and gentamicin protection assays. Caspase-1- and caspase-4-deficient cells showed significantly altered cytokine and chemokine profiles after infection with *A. actinomycetemcomitans,* showing reduced IL-17C and IL-18 release. We also found an increased release of TGF-α and LIF from caspase-4-deficient cells, along with elevated levels of the chemokines IL-8, CXCL9, and CXCL10. Additionally, both caspase-1- and caspase-4-deficient cells showed increased bacterial colonization and invasion, particularly in caspase-4-deficient cells. These findings suggest that caspase-1 and caspase-4 play distinct yet essential roles in gingival epithelial immunity, regulating cytokine release, barrier integrity, and defense against *A. actinomycetemcomitans* colonization.

## 1. Introduction

Periodontitis is a multifactorial inflammatory disease affecting the periodontium, which includes the gingiva, underlying connective tissue, cementum, alveolar bone, and the periodontal ligament [1]. Its development involves a complex interplay of microbial infection, genetic predisposition, environmental factors, and behavioral influences. The disease process is characterized by the disruption of the gingival epithelium, where subgingival pathogens interact with pattern-recognition receptors (PRRs) such as Toll-like receptors (TLRs) and NOD-like receptor (NLR), triggering an immune response [2,3,4].

*Aggregatibacter actinomycetemcomitans* (*A. actinomycetemcomitans*), a facultative Gram-negative and capnophilic periodontopathogen, is a significant component of the oral flora with the potential to cause aggressive systemic complications, including endocarditis and abscess formation in the lungs, head, neck, and abdominal regions [5]. One of the most important virulence factors of *A. actinomycetemcomitans* is leukotoxin. This membrane-active toxin specifically targets white blood cells, promoting cell lysis and contributing to the bacterium’s pathogenicity [6,7]. The leukotoxic activity of *A. actinomycetemcomitans* is strain-specific, with the JP2 clone, a highly leukotoxic variant, closely associated with the onset of attachment loss in adolescents. In addition to its cell-lytic properties, leukotoxin has been shown to stimulate the release of lysosomal enzymes and pro-inflammatory cytokines, amplifying the host inflammatory response [8,9].

A key component of the inflammatory response in periodontitis is the production and release of IL-1β, a pro-inflammatory cytokine. IL-1β secretion occurs through the activation of inflammasomes, multiprotein complexes that assemble in host cells upon recognition of microbial or danger-associated signals. This process involves the activation of caspase-1 and caspase-4, which play central roles in the maturation and release of bioactive IL-1β. Caspase-1, activated through the canonical NLRP3 inflammasome pathway, processes pro-IL-1β into its active form, while caspase-4, part of the non-canonical inflammasome pathway, is directly activated by intracellular lipopolysaccharides (LPS) from pathogens like *A. actinomycetemcomitans* [4,10,11,12,13,14].

Clinical and experimental evidence strongly support the involvement of caspase-1 and caspase-4 in the pathogenesis of periodontitis. Earlier studies have demonstrated that *A. actinomycetemcomitans* induces NLRP3-dependent IL-1β release in gingival epithelial cells [10,11,12]. Moreover, the bacterium’s leukotoxin has been implicated in inflammasome activation, triggering the release of IL-1β via both caspase-1 and caspase-4 pathways. The JP2 clone, with its heightened leukotoxic activity, induces particularly robust IL-1β responses, correlating with the severe tissue destruction observed in aggressive periodontitis [11]. However, currently, there is limited understanding of the role caspase-1 and caspase-4 play in the release of additional inflammatory mediators and their involvement in facilitating epithelial colonization by *A. actinomycetemcomitans*.

In the current study, we aim to further elucidate the involvement of caspase-1 and caspase-4, in regulating the immune response to *A. actinomycetemcomitans* infection in gingival epithelial cells. By understanding the specific roles of these caspases, we seek to uncover potential therapeutic targets for mitigating inflammation and tissue damage in periodontitis.

## 2. Material and Methods

### 2.1. Cell Culture and Bacteria

Human gingival epithelial cell line Ca9-22 (JCRB, Japanese Collection of Research Bioresources Cell Bank, Osaka, Japan), and caspase-1-and caspase-4-deficient cells previously created [10] with the CRISPR/Cas9 system were used in this study. The caspase-1-and caspase-4-deficient cells were validated using western blots [10]. The gingival epithelial cells were grown in Dulbecco’s modified eagle medium (DMEM) complemented with 10% fetal bovine serum (FBS), 2 mM L-glutamine, and 1 mM non-essential amino acids (all from Thermo Fisher Scientific, Waltham, MA, USA) at 37 °C and 5% CO_2_ atmosphere. During infection, DMEM complemented with 2% FBS, 1 mM non-essential amino acids, and 2 mM L-glutamine was used. The *Aggregatibacter actinomycetemcomitans* strain HK1651 (serotype b), a member of the highly virulent JP2 clone lineage, was used in this study. HK1651 belongs to the highly virulent JP2 clone lineage and was cultured in trypticase soy broth with 0.6% yeast at 37 °C under static conditions for 48 h prior to the experiments.

### 2.2. Targeted Olink Protein Analysis

Gingival epithelial cells (Cas9 control cells, caspase-1-and caspase-4-deficient cells) were infected with HK1651 at MOI300 for 24 h and incubated at 37 °C with 5% CO_2_. Following infection, supernatants were collected, centrifuged at 5000× *g* for 5 min, and stored at −80 °C. They were then analyzed using proximity extension assay (PEA) technology. The Olink Inflammation Panel (Olink Bioscience AB, Uppsala, Sweden), which utilizes PEA technology, allows for the quantification of 92 inflammation-related proteins. Protein levels are presented as folds of unstimulated cells using normalized protein expression (NPX) data. Proteins with signals below the limit of detection (LOD) were excluded from further analysis. The LOD is determined individually for each Olink assay and sample plate. It is calculated based on the background signal, estimated from the negative controls included on each plate, plus three standard deviations. The standard deviation is assay-specific and established during product validation for each panel.

### 2.3. Measurement of LDH Release

Gingival epithelial cells (Cas9 control cells, caspase-1-deficient, and caspase-4-deficient cells) were infected with HK1651 at MOI 300 for 24 h and incubated at 37 °C with 5% CO_2_. After infection, supernatants were collected, centrifuged at 5000× *g* for 5 min, and stored at −80 °C. Cell viability was assessed using the Pierce Lactate Dehydrogenase (LDH) Cytotoxicity Assay (Thermo Fisher Scientific), following the manufacturer’s instructions. The LDH assay results were analyzed using the Cytation 3 plate reader (BioTek, Winooski, VT, USA), as previously described [15].

### 2.4. Proliferation Assay

Gingival epithelial cells (Cas9 controls, caspase-1-deficient, and caspase-4-deficient cells) were infected with HK1651 at MOI300 for 24 h and incubated at 37 °C with 5% CO_2_. The cells were washed once with PBS after stimulation. Gingival epithelial cells were then stained with 0.1% crystal violet (diluted in 20% methanol, Sigma-Aldrich, St. Louis, MO, USA) for 10 min at room temperature, followed by two washes with tap water. The cells were subsequently destained using 1% sodium dodecyl sulfate on a shaker at 500 rpm for 5 min and measured at 570 nm using the Cytation 3 plate reader.

### 2.5. Colonization Assay

Gingival epithelial cells (Cas9 controls, caspase-1-deficient, and caspase-4-deficient cells) were infected with HK1651 (FITC labeled) at MOI500 for 4 h and incubated at 37 °C with 5% CO_2_. The assay was conducted to evaluate bacterial colonization (adherent and intracellular bacteria) of gingival epithelial cells. We utilized a higher MOI in this assay to compensate for the shorter infection duration. The gingival epithelial cells were then washed ten times with PBS, and the fluorescence (FITC, Sigma-Aldrich) was measured and imaged using the Cytation 3 plate reader. Colonization is presented as mean fluorescence intensity (MFI).

### 2.6. Invasion Assay

Gingival epithelial cells (Cas9 controls, caspase-1-deficient, and caspase-4-deficient cells) were infected with HK1651 at MOI500 for 2 h and incubated at 37 °C with 5% CO_2_. We utilized a higher MOI in this assay to compensate for the shorter infection duration. The cells were then washed ten times with PBS. To kill the extracellular HK1651, DMEM with 100 μg/mL gentamicin was added to the gingival epithelial cells for 1 h. The plate was then washed three times and the gingival epithelial cells were lysed with 0.1% Triton-X 100 in PBS. HK1651 bacteria were then plated on TSA plates with 0.6% yeast and counted 24–48 h after incubation at 37 °C, as previously described [15].

### 2.7. Statistical Analysis

The presented data are shown as mean ± standard error of the mean (SEM). Differences between groups were assessed by unpaired Student’s *t*-tests or one-way ANOVA, followed by Bonferroni multiple testing corrections. The Shapiro–Wilk test was used to evaluate normality.

## 3. Results

### 3.1. Differentially Regulated Cytokines and Cytokine Receptors in Caspase-1-and Caspase-4-Deficient Cells

**Figure 1:**
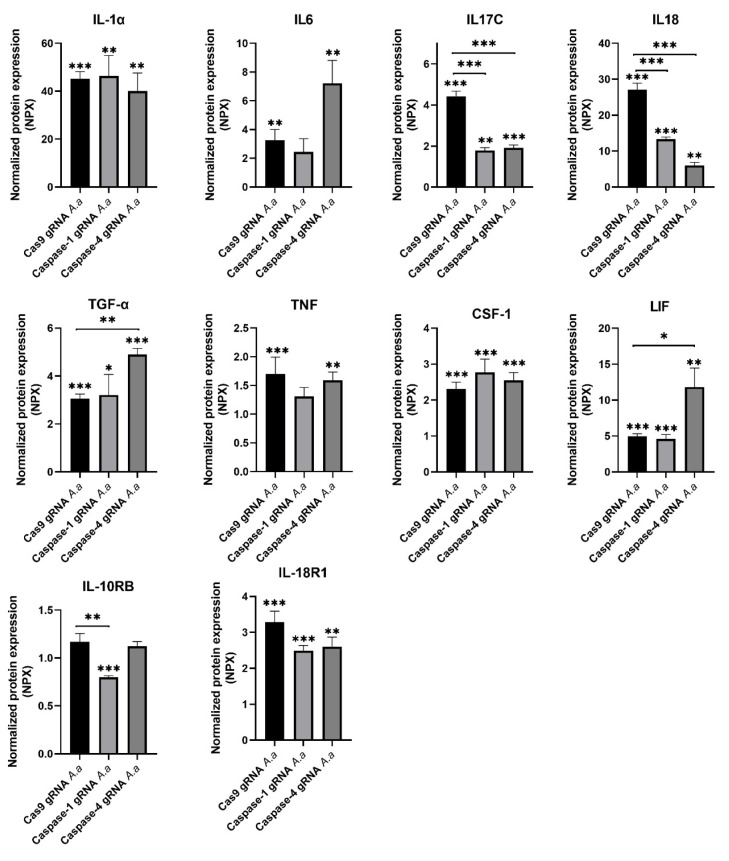
Cytokine release from caspase-1- and caspase-4-deficient cells. Gingival epithelial cells were infected with *A. actinomycetemcomitans* (*A. a*, MOI 300) for 24 h followed by analysis of cytokine release. Data are presented as mean ± SEM (n = 4 independent experiments). Asterisks denote statistical significance compared to Cas9 gRNA cells (* *p* < 0.05, ** *p* < 0.01, *** *p* < 0.001).

The gingival epithelial cells (Cas9 controls, caspase-1-deficient, and caspase-4-deficient cells) were infected with HK1651 for 24 h and the cytokine and cytokine receptor release were evaluated. The expression of ten proteins (IL-1-α, IL-6, IL-17C, IL-18, TGF-α, TNF, CSF-1, LIF, IL-10RB, and IL-18R1) was identified as being significantly altered after HK1651 infection compared to unstimulated Cas9 and/or caspase1- and caspase-4-deficient cells (Figure 1). Of the ten proteins, five of these proteins secreted by caspase1- and caspase-4-deficient cells differed significantly in comparison to Cas9 cells after infection. The protein expression of three proteins (IL-17C, IL-18, and IL-10RB) was significantly decreased in caspase-1-deficient cells compared to Cas9 cells following HK1651 infection (Figure 1). The protein expression of two proteins (IL-17C and IL-18) was significantly decreased and the protein expression of two proteins (TGF-α and LIF) was significantly increased in caspase-4-deficient cells compared to Cas9 cells following HK1651 infection (Figure 1). Taken together, our findings highlight that caspase-1 and caspase-4 are important for regulating cytokine release from gingival epithelial cells.

### 3.2. Chemokine Regulation in Caspase-1-and Caspase-4-Deficient Cells

**Figure 2:**
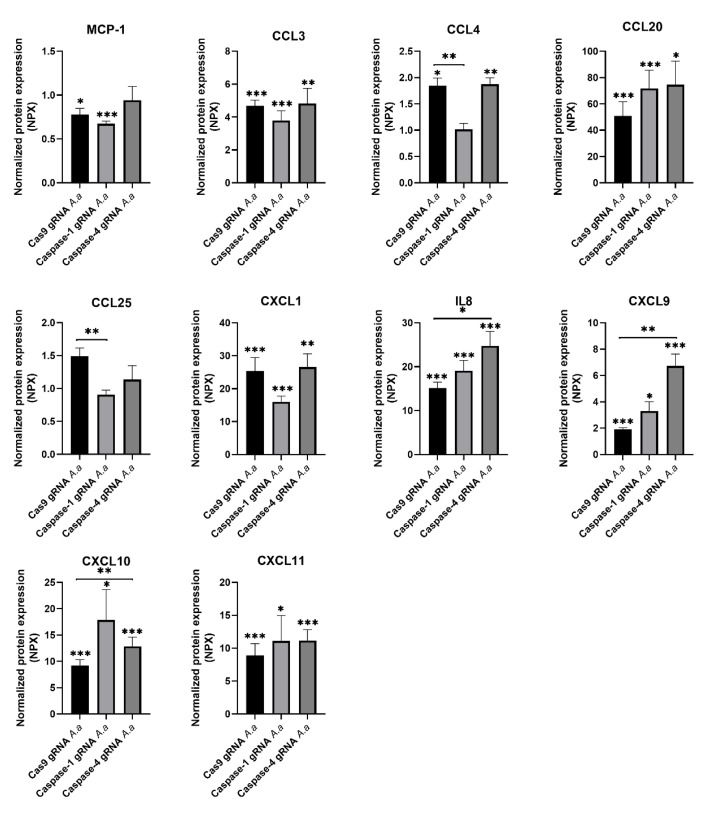
Chemokine release from caspase-1- and caspase-4-deficient cells. Gingival epithelial cells were infected with *A. actinomycetemcomitans* (*A. a*, MOI 300) for 24 h followed by analysis of chemokine release. Data are presented as mean ± SEM (n = 4 independent experiments). Asterisks denote statistical significance compared to Cas9 gRNA cells (* *p* < 0.05, ** *p* < 0.01, *** *p* < 0.001).

We continued to investigate the role of caspase-1 and caspase-4 in the release of chemokines from gingival epithelial cells after infection with HK1651 for 24 h. The expression of nine proteins (MCP-1, CCL3, CCL4, CCL20, CXCL1, IL-8, CXCL9, CXCL10, and CXCL11) were identified as being significantly altered after HK1651 infection compared to unstimulated Cas9 and/or caspase1- and caspase-4-deficient cells (Figure 2). Of the nine proteins, five of these proteins secreted by caspase-1- and caspase-4-deficient cells differed significantly in comparison to Cas9 cells after infection. The protein expression of two proteins (CCL4 and CCL25) significantly decreased in caspase-1-deficient cells compared to Cas9 cells following HK1651 infection (Figure 2). The protein expression of three proteins (IL-8, CXCL9, and CXCL10) were significantly increased in caspase-4-deficient cells compared to Cas9 cells following HK1651 infection (Figure 2). Taken together, our findings showed that caspase-1 and caspase-4 can regulate chemokine release from gingival epithelial cells.

### 3.3. Differentially Regulated Inflammatory Mediators in Caspase-1-and Caspase-4-Deficient Cells

**Figure 3:**
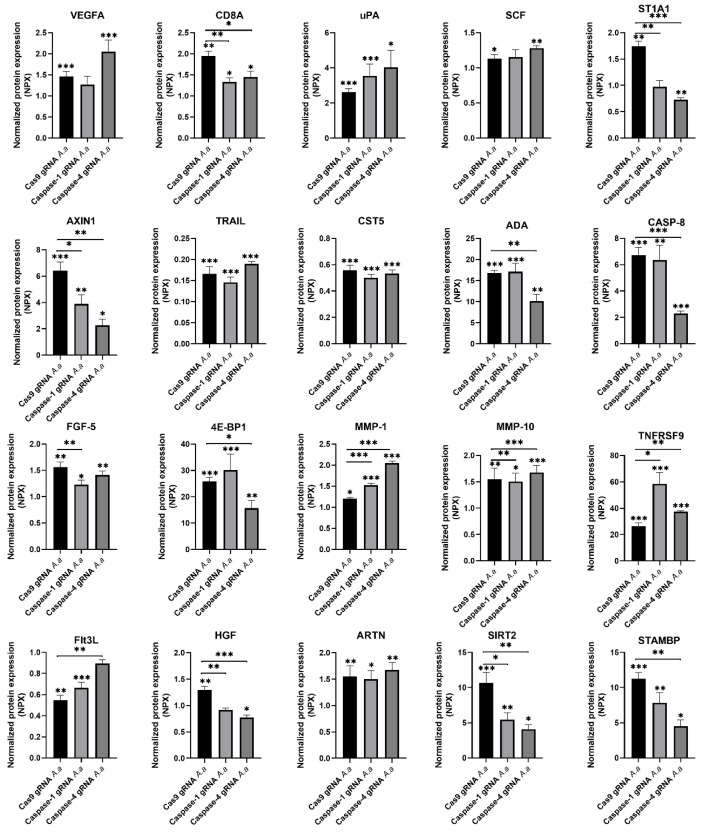
Inflammatory mediator release from caspase-1- and caspase-4-deficient cells. Gingival epithelial cells were infected with *A. actinomycetemcomitans* (*A. a*, MOI 300) for 24 h followed by analysis of inflammatory mediator release. Data are presented as mean ± SEM (n = 4 independent experiments). Asterisks denote statistical significance compared to Cas9 gRNA cells (* *p* < 0.05, ** *p* < 0.01, *** *p* < 0.001).

Next, we continued to investigate the role of caspase-1 and caspase-4 in the release of additional inflammatory mediators from gingival epithelial cells after infection with HK1651 for 24 h. The expression of 20 proteins (VEGFA, CD8A, uPA, SCF, ST1A1, AXIN1, TRAIL, CST5, ADA, CASP-8, FGF-5, 4E-BP1, MMP-1, MMP-10, TNFRSF9, Flt3L, HGF, ARTN, SIRT2, and STAMBP) were identified as being significantly altered after HK1651 infection compared to unstimulated Cas9 and/or caspase-1- and caspase-4-deficient cells (Figure 3). Of the 20 proteins, 14 of these proteins secreted by caspase-1-and caspase-4-deficient cells differed significantly in comparison to Cas9 cells after infection. The protein expression of seven proteins (CD8A, ST1A1, AXIN1, FGF-5, MMP-10, HGF, SIRT2) was significantly decreased and the protein expression of two proteins (MMP-1, TNFRSF9) was significantly increased in caspase-1-deficient cells compared to Cas9 cells following HK1651 infection (Figure 3). The protein expression of nine proteins (CD8A, ST1A1, AXIN1, ADA, CASP-8, 4E-BP1, HGF, SIRT2, STAMBP) was significantly decreased and the protein expression of four proteins (MMP-1, MMP-10, TNFRSF9, Flt3L) was significantly increased in caspase-4-deficient cells compared to Cas9 cells following HK1651 infection (Figure 3). Taken together, our findings highlight that caspase-1 and caspase-4 are important for regulating the release of inflammatory mediators from gingival epithelial cells.

### 3.4. Altered Proliferation and Cell Viability in Caspase-1-and Caspase-4-Deficient Cells

**Figure 4:**
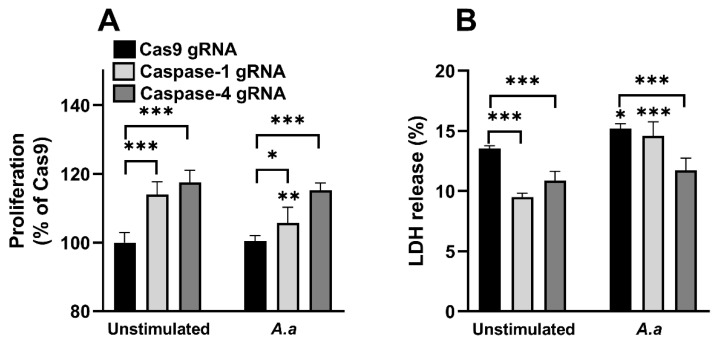
Increased proliferation and cell viability. Gingival epithelial cells were infected with *A. actinomycetemcomitans* (*A. a*, MOI 300) for 24 h followed by analysis of cell proliferation (**A**) and LDH release (**B**). Data are presented as mean ± SEM (n = 4 independent experiments). Asterisks denote statistical significance compared to unstimulated cells (* *p* < 0.05, ** *p* < 0.01, *** *p* < 0.001).

We proceeded with investigating the proliferation and cell viability of the gingival epithelial cells (Cas9 controls, caspase-1- and caspase-4-deficient cells) after HK1651 stimulation. We found that the caspase-1- and caspase-4-deficient cells exhibited significantly increased proliferation compared to Cas9 cells during unstimulated conditions and during HK1655 infection (Figure 4A). We also found that HK1655 reduced the proliferation of caspase-1-deficient cells compared to unstimulated caspase-1-deficient cells (Figure 4A). Next, we continued with evaluating the cell viability through LDH release. We found that the caspase-1- and caspase-4-deficient cells released significantly less LDH compared to Cas9 cells during unstimulated conditions (Figure 4B). We also found that HK1651 significantly increased the LDH release in Cas9 and caspase-1-deficient cells, but not in caspase-4-deficient cells compared to unstimulated cells (Figure 4B). Taken together, our findings showed that caspase-1 and caspase-4 are important for gingival epithelial cell viability and proliferation.

### 3.5. Caspase-1 and Caspase-4 Protects Gingival Epithelial Cells Against Colonization

**Figure 5:**
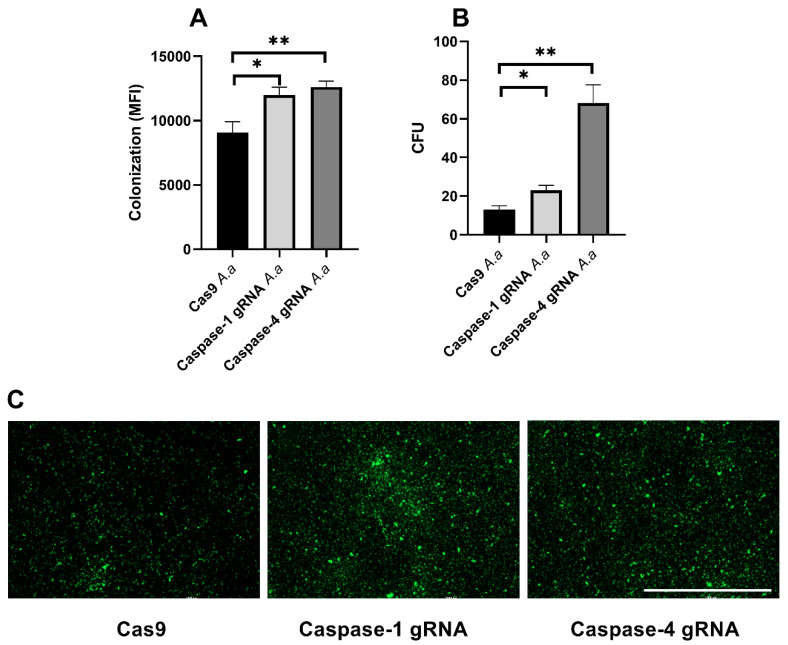
Increased bacterial colonization and invasion. Gingival epithelial cells were infected with *A. actinomycetemcomitans* (*A. a*) for 4 (**A**,**C**) or 2 (**B**) h followed by evaluation of colonization (**A**,**C**) and invasion (**B**). *A. actinomycetemcomitans* (FITC labeled) colonization was quantified as mean fluorescence intensity (MFI) (**A**) and imaged (**C**). Data are presented as mean ± SEM of n = 3–5 independent experiments. The asterisk shows statistical significance (* *p* < 0.05, ** *p* < 0.01). Scale bar: 1000 µm.

We proceeded to assess the roles of caspase-1 and caspase-4 in the colonization and invasion of gingival epithelial cells by HK1651. We found that the bacterial colonization (bacterial colonization reflects a combination of attached extracellular bacteria and intracellular bacteria) in caspase-1-and caspase-4-deficicent cells was significantly increased compared to Cas9 cells (Figure 5A). This was also validated by the imaging of the FITC-labeled bacteria (Figure 5C). To specifically assess whether caspase-1 and caspase-4 deficiency affects the ability of HK1655 to invade gingival epithelial cells, the number of intracellular HK1651 was quantified. The bacterial invasion of caspase-1- and caspase-4-deficient cells was significantly increased compared to the invasion of Cas9 cells (Figure 5B). The highest colonization and invasion levels were observed for caspase-4-deficient cells (Figure 5A,B). Taken together, our findings highlight that caspase-1 and caspase-4 are important for protecting gingival epithelial cells from *A. actinomycetemcomitans* adhesion and invasion.

## 4. Discussion

This study provides new insights into the roles of caspase-1 and caspase-4 in regulating gingival epithelial cell responses to *A. actinomycetemcomitans* infection. Our results indicate that caspase-1 and caspase-4 not only contribute to immune response regulation but also serve as critical regulators of host defense mechanisms against bacterial colonization and invasion.

A key finding from our study was the differential release of cytokines and chemokines from caspase-1- and caspase-4-deficient cells following *A. actinomycetemcomitans* infection. The significant reduction in IL-17C and IL-18 release from both caspase-1- and caspase-4-deficient cells underscores a common regulatory role of these caspases in inflammasome activation and cytokine release. IL-17C, primarily produced by epithelial cells, plays an important role in the host response by enhancing epithelial immunity and barrier integrity [16,17]. Similarly, IL-18, a well-established inflammasome-activated cytokine [13,14], is linked to immune cell recruitment and inflammatory responses in periodontitis [18]. We also found a significant increase in TGF-α and LIF release in caspase-4-deficient gingival epithelial cells following *A. actinomycetemcomitans* infection. Previous studies have shown that TGF-α is essential for wound healing and epithelial cell proliferation, both of which are critical for maintaining barrier integrity during inflammation [19]. Notably, TGF-α levels are significantly reduced in patients with periodontal disease [20], supporting the notion that caspase-4 regulates epithelial responses in disease progression. Also, LIF has been implicated in periodontal tissue remodeling and inflammation, with increased expression observed in periodontal tissues [21]. LIF plays a role in immune modulation and epithelial homeostasis [22], further indicating its involvement in periodontal pathogenesis. The increased release of both TGF-α and LIF in caspase-4-deficient cells suggests that caspase-4 functions to limit excessive epithelial proliferation and regulate tissue remodeling. This finding highlights the potential role of caspase-4 in active periodontitis, where dysregulated repair mechanisms may contribute to disease progression. Taken together, the altered cytokine release from the caspase-deficient cells strengthens the notion that caspase-1 and caspase-4 are important for cytokine regulation in gingival epithelial cells.

Chemokines play a crucial role in recruiting immune cells to the site of infection and inflammation [23]. In this study, we found that caspase-4-deficient cells exhibited a significant increase in IL-8, CXCL9, and CXCL10 release. IL-8 is a potent chemoattractant for neutrophils, and CXCL9 and CXCL10 facilitate the recruitment of activated T cells and NK cells [23]. The elevated levels of these chemokines in the absence of caspase-4 suggest that caspase-4 plays a regulatory role in limiting excessive immune recruitment, preventing hyperinflammatory responses during infection. Conversely, the downregulation of CCL4 and CCL25 in caspase-1-deficient cells suggests that caspase-1 is essential for maintaining adequate recruitment of macrophages and dendritic cells, which are important for mounting an effective immune response against bacterial infections [24,25]. While both caspase-1 and caspase-4 regulate chemokine responses, their roles appear to be distinct but complementary. Caspase-1 primarily contributes to early innate immune responses by facilitating the recruitment of macrophages and dendritic cells, which are essential for antigen presentation and bacterial clearance. On the other hand, caspase-4 influences both innate and adaptive immunity by modulating the recruitment of neutrophils and T cells.

Our study also identified several inflammatory mediators that were differentially regulated in caspase-deficient cells. Caspase-1-deficient cells exhibited reduced secretion of tissue repair-associated proteins such as HGF, MMP-10, and FGF-5. These proteins play essential roles in epithelial proliferation, extracellular matrix remodeling, and wound healing. The reduced expression of these mediators suggests that caspase-1 may not only regulate inflammatory cytokine responses but also facilitate tissue repair during bacterial infections [26]. Interestingly, caspase-1- and caspase-4-deficient cells displayed increased release of TNFRSF9, a receptor involved in T-cell activation and immune regulation [27], which may indicate a compensatory upregulation of immune signaling pathways aimed at balancing immune responses in the absence of caspase-1 and caspase-4. Caspase-4 also appears to have a significant role in apoptosis and metabolic regulation. The downregulation of caspase-8 in caspase-4-deficient cells suggests impaired apoptotic pathways, which could delay inflammation resolution and contribute to prolonged immune responses [28]. Similarly, the reduced expression of ADA and SIRT2, both involved in cellular senescence and inflammation [29,30], emphasizes the importance of caspase-4 in maintaining cell death and metabolic homeostasis during bacterial infections. Furthermore, STAMBP, which is known to regulate inflammasome activation and IL-1β secretion in monocytes, acting as a negative regulator of the NLRP3 inflammasome [31], was reduced in caspase-4-deficient gingival epithelial cells. This suggests that caspase-4 may control inflammasome activation, potentially preventing excessive inflammatory damage. Taken together, these findings strengthen the notion that caspase-1 and caspase-4 are key players regulating the immune response during an *A. actinomycetemcomitans* infection.

Our findings suggest that caspase-1 and caspase-4 play critical roles in modulating the immune response beyond their conventional enzymatic activity. A previous study has demonstrated that enzymatically inactive caspase-1 can still mediate inflammation by enhancing the activation of NF-κB, independent of its proteolytic function. This highlights a potential scaffolding role for caspase-1 in inflammatory signaling [32]. Similarly, emerging evidence indicates that caspase-4 interacts with additional inflammatory pathways, further supporting its role as an immune regulator. Notably, caspase-4 has been shown to associate with TNF receptor-associated factor 6 (TRAF6), a key adapter protein involved in NF-κB signaling [33]. This interaction facilitates NF-κB activation, leading to an increased transcription of pro-inflammatory mediators. By influencing these pathways, caspase-4 may contribute to sustained inflammatory responses, further amplifying immune activation in response to infection. These findings suggest that both caspase-1 and caspase-4 serve dual functions as both conventional mediators of inflammasome activation and regulators of broader inflammatory signaling networks. Hence, these functions may contribute to the underlying mechanisms responsible for the altered inflammatory mediator release from caspase-1- and caspase-4-deficient cells.

An important finding in our study is the protective role of caspase-1 and caspase-4 in preventing *A. actinomycetemcomitans* colonization and invasion of gingival epithelial cells. Both caspase-1- and caspase-4-deficient cells exhibited significantly higher bacterial adherence and intracellular invasion compared to Cas9 control cells, with the highest invasion observed in caspase-4-deficient cells. This suggests that caspase-4 plays a particularly crucial role in maintaining epithelial integrity and preventing bacterial dissemination. Previous studies have established that inflammasome activation leads to the induction of pyroptosis, a form of programmed cell death that eliminates infected cells to limit bacterial dissemination [28]. The increased survival and proliferation of caspase-deficient cells, coupled with their reduced LDH release, suggest an impairment in pyroptotic cell death. The accumulation of viable, non-pyroptotic cells may hinder intracellular bacterial clearance while promoting bacterial adherence and invasion, as shown by the increased colonization observed in caspase-deficient cells.

In conclusion, this study demonstrates that caspase-1 and caspase-4 play essential and distinct roles in regulating gingival epithelial immune responses during *A. actinomycetemcomitans* infection. Both caspase-1 and caspase-4 are important for cytokine and chemokine release, epithelial barrier maintenance, and protection against bacterial colonization and invasion. Understanding the specific contributions of caspase-1 and caspase-4 in periodontitis pathogenesis could pave the way for novel therapeutic interventions aimed at restoring immune homeostasis and preventing disease progression.

## Data Availability

Data are contained within the article.
